# Three dimensional palatal morphology and dentoalveolar differences after extraction and non extraction treatment in class II malocclusion

**DOI:** 10.1038/s41598-026-37842-y

**Published:** 2026-01-30

**Authors:** Meliha Rübendiz, Ezgi Kardelen Altunal, Merve Berika Kadıoğlu, Meyra Durmaz

**Affiliations:** 1 Department of Orthodontics, Faculty of Dentistry, Ankara University, 06500 Ankara, Turkey; 2 Department of Orthodontics, The Graduate School of Health Sciences of Ankara University, 06110 Ankara, Turkey

**Keywords:** Digital orthodontics, 3D digital models, Class II malocclusion, Extraction vs non-extraction, Palatal morphology, Palatal volume and surface area, Cephalometric analysis, Anatomy, Diseases, Health care, Medical research

## Abstract

Digital technologies have transformed orthodontics by enabling precise three-dimensional (3D) assessment of treatment outcomes; however, the effects of extraction versus non-extraction strategies on palatal morphology in Class II malocclusions remain underexplored. This study quantified segmental palatal volume and surface changes, along with skeletal–dentoalveolar outcomes, using digital model analysis integrated with cephalometrics. Records of 69 post-pubertal patients treated with fixed appliances (Class II, n = 34; Class I controls, n = 35) were retrospectively analyzed, each subdivided into extraction and non-extraction protocols. Maxillary casts were scanned with Sirona inEos X5 and segmented in Materialise 3-Matic to compute palatal volume and surface area, while cephalometric variables were derived from lateral radiographs (Dolphin Imaging). Group comparisons were performed using non-parametric tests (*p* < 0.05). Multivariable regression analyses were conducted to identify dentoalveolar predictors of palatal remodeling. Intra-examiner repeatability was excellent, as indicated by a high intraclass correlation coefficient (ICC ≥ 0.90). Skeletal changes were minimal and comparable across groups. Dentoalveolar findings demonstrated significant maxillary incisor protrusion in non-extraction protocols and retrusion in extraction protocols (*p* < 0.05). Anterior palatal volume and surface area increased across all groups. Overall, measurable differences in total palatal volume and surface area were observed between extraction and non-extraction treatment strategies. Regression analyses indicated that sagittal dentoalveolar displacement was associated with palatal remodeling, whereas angular and vertical dental variables showed limited independent influence. Extraction and non-extraction treatment protocols were associated with measurable differences in three-dimensional palatal volume and surface area, reflecting distinct dentoalveolar and palatal remodeling patterns between treatment strategies.

**Supplementary Information:**

The online version contains supplementary material available at 10.1038/s41598-026-37842-y.

## Introduction

Class II malocclusions, characterized by varying combinations of skeletal and dental discrepancies, are among the most frequently encountered orthodontic problems. The treatment procedure is influenced by etiological determinants, the severity of the malocclusion, the patient’s age, and professional experience of clinician^1^. Treatment strategies for growing patients generally focus on growth-modification, while surgical approaches are preferred for adults with more severe malocclusions^2^. However adults with mild to moderate malocclusions, management generally involves fixed appliance therapy, implemented with or without extractions^3^.

The decision to extract teeth for orthodontic treatment has long been a challenging issue for orthodontists in their daily practice^4^. In orthodontic treatments, in addition to eliminating crowding and achieving ideal incisor positions, the goal is to provide acceptable occlusion by establishing harmony between dental arches in the sagittal, vertical, and transverse directions^5^. In addition to those who advocate tooth extraction in borderline cases^6^, there are also researchers who recommend developing dental arches instead of extracting teeth for orthodontic purposes^7^.

Class II cases involving factors such as severe crowding, Bolton discrepancies, protrusive incisor positions, bimaxillary protrusion, midline deviations, and the presence of teeth with endodontic or periodontal problems are also considered indications for orthodontic extraction^6^. However, in the non-extraction treatment of Class II malocclusion, maxillary expansion and/or distalization are generally recommended, with mesialization and/or expansion of the mandibular dental arch when necessary^8^.

Dentoalveolar adaptations occur in both extraction and non-extraction treatments and can influence post-treatment stability. In addition, these therapeutic approaches may alter palatal morphology^9–11^. While conventional cephalometric analyses provide information on skeletal and dentoalveolar changes, they do not fully capture three-dimensional (3D) alterations in palatal morphology, which are relevant to tongue posture, oral function, post-treatment stability, and relapse risk^12,13^. In this context, although previous studies have compared extraction and non-extraction strategies with respect to palatal morphology, most of the evidence is still based on two-dimensional assessments^9^. While some investigations have utilized 3D digital models, data specifically addressing the effects of extraction versus non-extraction approaches in Class II malocclusions remain limited^14^. Digital model analysis now allows accurate, reproducible volumetric and surface quantification, offering new perspectives on functional outcomes^15–17^.

Functional factors may also contribute to variations in palatal morphology and should be considered when interpreting three-dimensional palatal outcomes. In particular, mouth breathing and oral habits have been shown to influence palatal width, height, and overall maxillary morphology through alterations in tongue posture and perioral muscular balance. These functional adaptations may affect transverse and vertical palatal development, potentially contributing to interindividual differences in palatal form. Although breathing patterns and oral habits were not directly evaluated in the present retrospective study, acknowledging their potential influence provides a broader biological and functional context for understanding palatal remodeling associated with different orthodontic space-management strategies^18,19^.

These methodological advances underline the need to interpret morphological outcomes not only as dimensional changes but also within a broader clinical context. Alterations in palatal morphology have been associated with changes in oral cavity equilibrium and tongue posture in previous studies^12,20,21^; however, such functional implications were not directly evaluated in the present study and should therefore be interpreted with caution. While reductions in palatal volume following extraction therapy have been suggested to influence intraoral spatial relationships, the present findings are limited to morphological assessments. Hence, evaluating palatal morphology in conjunction with skeletal and dentoalveolar parameters may contribute to a more comprehensive understanding of treatment-related morphological adaptations.

Although previous studies have examined extraction and non-extraction orthodontic treatments primarily with respect to skeletal and dentoalveolar outcomes, the available evidence on palatal morphology remains limited^11,14^. .Most existing studies rely on two-dimensional assessments or isolated linear measurements, and comprehensive three-dimensional analyses quantifying palatal surface area and volume are scarce^11,22^. In particular, data addressing three-dimensional palatal changes in Class II malocclusions treated with extraction versus non-extraction protocols, together with their associated dentoalveolar adaptations, remain largely unaddressed in the literature.

Therefore, the present study aimed not only to compare skeletal and dentoalveolar changes but also to investigate three-dimensional alterations in palatal morphology—including surface area and volume—in Class II patients treated with extraction and non-extraction protocols, using Class I individuals as a control group.

The null hypothesis was that there would be no significant differences in three-dimensional palatal volume, surface area, or dentoalveolar parameters between extraction and non-extraction treatment protocols in post-pubertal Class II patients.

## Results

Baseline (T1) intergroup comparisons of skeletal, dentoalveolar, volumetric, and surface-area parameters—along with chronological age (CA), skeletal maturation (SM), arch length discrepancy (ALD), and treatment duration (TD)—are summarized in Table 1. Intra-examiner reliability was excellent across all variables, with intraclass correlation coefficient (ICC) values ≥ 0.90 (Supplementary Table S1).

**Table 1:** Baseline (T1) intergroup comparisons of skeletal, dentoalveolar, and palatal volumetric/surface-area parameters, together with chronological age (CA), skeletal maturation (SM), arch length discrepancy (ALD), and treatment duration (TD).

	Class II		Class I				
Variables	Non-Extraction (1)	Extraction (2)	Non-Extraction (3)	Extraction (4)	Test KW	Mann–Whitney U	
	X̅ ± SE	X̅ ± SE	X̅ ± SE	X̅ ± SE	p	1–2	3–4
SNA (°)	81.39 ± 0.59	80.19 ± 0.66	81.57 ± 0.66	79.51 ± 0.74	ns	-	-
SNB (°)	76.04 ± 0.63	74.87 ± 0.7	79.81 ± 0.65	77.25 ± 0.76	*	ns	*
ANB (°)	5.34 ± 0.24	5.32 ± 0.28	1.82 ± 0.24	2.28 ± 0.29	ns	-	-
SN/GoGn (°)	34.53 ± 1.09	34.82 ± 1.3	31.78 ± 1.09	34.47 ± 1.12	ns	-	-
U1/NA (°)	18.02 ± 2.34	19.26 ± 1.31	24.12 ± 0.84	23.59 ± 1.64	ns	-	-
U1⊥NA (mm)	2.8 ± 0.45	2.95 ± 0.46	4.36 ± 0.29	4.41 ± 0.51	ns	-	-
L1/NB (°)	30.15 ± 1.16	30.33 ± 0.96	26.18 ± 0.7	25.47 ± 1.29	ns	-	-
L1⊥NB (mm)	4.83 ± 0.41	5.38 ± 0.28	3.58 ± 0.2	4 ± 0.45	ns	-	-
Overjet (mm)	4.05 ± 0.35	4.07 ± 0.31	2.75 ± 0.25	2.97 ± 0.23	ns	-	-
Overbite (mm)	2.67 ± 0.39	2.18 ± 0.27	2.18 ± 0.26	2.34 ± 0.35	ns	-	-
V3 (cm^3^)	0.27 ± 0.04	0.19 ± 0.02	0.24 ± 0.02	0.15 ± 0.02	*	*	*
V4 (cm^3^)	1.27 ± 0.07	0.97 ± 0.07	1.36 ± 0.11	0.97 ± 0.06	*	*	*
V5 (cm^3^)	1.94 ± 0.08	1.94 ± 0.09	2.13 ± 0.1	1.93 ± 0.07	ns	-	-
V6 (cm^3^)	3.51 ± 0.17	3.45 ± 0.17	3.71 ± 0.18	3.59 ± 0.16	ns	-	-
TV (cm^3^)	6.99 ± 0.29	6.51 ± 0.31	7.49 ± 0.36	6.65 ± 0.22	ns	-	-
A3 (cm^2^)	2.24 ± 0.21	1.72 ± 0.18	2.24 ± 0.23	1.56 ± 0.15	*	*	*
A4 (cm^2^)	3.72 ± 0.14	3.3 ± 0.13	4.45 ± 0.25	3.34 ± 0.14	*	*	*
A5 (cm^2^)	3.41 ± 0.12	3.71 ± 0.16	3.75 ± 0.14	3.64 ± 0.13	ns	-	-
A6 (cm^2^)	5.43 ± 0.23	5.47 ± 0.25	5.41 ± 0.24	5.53 ± 0.27	ns	-	-
TSA (cm^2^)	14.24 ± 0.34	14.16 ± 0.41	15.24 ± 0.45	13.84 ± 0.37	ns	-	-
CA (year)	13.8 ± 1.3	15.68 ± 0.72	16.55 ± 1.51	16.08 ± 0.8	ns	-	-
SM (%)	98.96 ± 0.44	98.87 ± 0.45	98.47 ± 0.46	98.39 ± 0.6	ns	-	-
TD (month)	25.78 ± 2.61	25.88 ± 1.69	19 ± 1.73	32.59 ± 1.92	*	ns	*
ALD (mm)	-4.03 ± 0.59	-6.15 ± 0.91	-2.92 ± 0.8	-6.73 ± 0.71	*	*	*

X̅: mean; SE: standard error; KW: Kruskal–Wallis analysis; *: p < 0.05; ns: not significant; (–): not applied; TV: total volume; TSA: total surface area; CA: chronological age; SM: percentage of skeletal maturation; TD: treatment duration; ALD: arch length discrepancy.

Chronological age and skeletal maturation were comparable across all four subgroups. However, significant intergroup differences were detected in ALD and TD (*p* < 0.05). Further analysis showed that ALD differences originated from lower values in the non-extraction groups, whereas longer treatment times were observed in the extraction subgroups of the control group. Pre-treatment evaluation of skeletal and dentoalveolar variables revealed significant differences only in the SNB angle within control subgroups (p < 0.05). Baseline intergroup comparisons of palatal volume and surface-area parameters did not reveal statistically significant differences (Kruskal–Wallis, p > 0.05); however, baseline measurements were not considered indicative of equivalence and were accounted for by focusing on treatment-related change scores (ΔT2–T1).

Post-treatment evaluation indicated no significant intergroup differences in skeletal parameters (Table 2). However, significant differences were observed in the sagittal and angular positions of maxillary and mandibular incisors (*p* < 0.05), which were attributable to extraction versus non-extraction strategies in both Class II and Class I cohorts. For sagittal positional changes of the maxillary incisors and molars (U1⊥VRL and U6⊥VRL), significant intergroup differences were observed (*p* < 0.05), mainly arising from extraction–non-extraction contrasts. In contrast, no significant differences were detected in vertical positional changes of the maxillary incisors and molars (U1⊥SN, U6⊥SN).

**Table 2:** Intragroup (ΔT2–T1, Wilcoxon signed-rank) and intergroup (Kruskal–Wallis and Mann–Whitney U) comparisons of skeletal, dentoalveolar, and palatal volumetric/surface-area outcomes.

	Class II				Class I				Test KW	Mann–Whitney U			
	Non-Extraction (1)	W	Extraction (2)	W	Non-Extraction (3)	W	Extraction (4)	W		1–2	3–4	1–3	2–4
	X̅ ± SE Δ (T2–T1)		X̅ ± SE Δ (T2–T1)		X̅ ± SE Δ (T2–T1)		X̅ ± SE Δ (T2–T1)						
SNA (°)	0.01 ± 0.18	ns	-0.23 ± 0.17	ns	-0.17 ± 0.12	ns	-0.28 ± 0.14	ns	ns	-	-	-	-
SNB (°)	0.13 ± 0.18	ns	-0.04 ± 0.18	ns	-0.13 ± 0.19	ns	-0.25 ± 0.15	ns	ns	-	-	-	-
ANB (°)	-0.07 ± 0.08	ns	-0.14 ± 0.14	ns	0.15 ± 0.1	ns	-0.04 ± 0.1	ns	ns	-	-	-	-
SN/GoGn (°)	-0.09 ± 0.17	ns	-0.05 ± 0.19	ns	-0.23 ± 0.22	ns	-0.22 ± 0.28	ns	ns	-	-	-	-
U1/NA (°)	3.64 ± 2.83	ns	-2.62 ± 1.64	ns	3.64 ± 1.13	*	-3.29 ± 1.67	*	*	*	*	ns	ns
U1⊥NA (mm)	0.87 ± 0.46	ns	-0.63 ± 0.34	ns	0.97 ± 0.26	*	-1.17 ± 0.42	*	*	*	*	ns	ns
L1/NB (°)	5.33 ± 1.67	*	-0.79 ± 1.37	ns	3.82 ± 1.12	*	-0.84 ± 1.21	ns	*	*	*	ns	ns
L1⊥NB (mm)	1.94 ± 0.37	*	-0.11 ± 0.37	ns	1.12 ± 0.36	*	-0.25 ± 0.35	ns	*	*	*	ns	ns
U1⊥VRL (mm)	0.82 ± 0.56	ns	-1.15 ± 0.56	ns	1.27 ± 0.35	*	-1.07 ± 0.49	ns	*	*	*	ns	ns
U6D⊥VRL (mm)	-0.92 ± 0.47	*	1.74 ± 0.23	*	-0.29 ± 0.27	ns	2.47 ± 0.44	*	*	*	*	*	ns
U1⊥SN (mm)	0.7 ± 0.15	*	0.55 ± 0.14	*	0.91 ± 0.28	*	0.9 ± 0.27	*	ns	-	-	-	-
U6⊥SN (mm)	0.58 ± 0.14	*	0.79 ± 0.15	*	0.67 ± 0.14	*	1.12 ± 0.27	*	ns	-	-	-	-
Overjet (mm)	-1.34 ± 0.27	*	-1.73 ± 0.25	*	-0.49 ± 0.15	*	-0.68 ± 0.26	*	*	ns	ns	*	*
Overbite (mm)	-0.09 ± 0.27	ns	0.21 ± 0.16	ns	0.36 ± 0.24	ns	0.24 ± 0.27	ns	ns	-	-	-	-
V3 (cm^3^)	0.15 ± 0.04	*	0.36 ± 0.05	*	0.17 ± 0.04	*	0.43 ± 0.05	*	*	*	*	ns	ns
V4 (cm^3^)	0.21 ± 0.09	*	-	-	0.17 ± 0.08	ns	-	-	ns	-	-	-	-
V5 (cm^3^)	0.2 ± 0.06	*	-0.11 ± 0.08	ns	0.22 ± 0.06	*	0.03 ± 0.08	ns	*	*	*	ns	ns
V6 (cm^3^)	0.22 ± 0.11	ns	0.12 ± 0.08	ns	0.41 ± 0.12	*	0.16 ± 0.1	ns	ns	-	-	-	-
TV (cm^3^)	0.86 ± 0.16	*	-0.57 ± 0.17	*	0.94 ± 0.19	*	-0.37 ± 0.14	*	*	*	*	ns	ns
A3 (cm^2^)	0.81 ± 0.24	*	1.63 ± 0.24	*	0.84 ± 0.23	*	1.94 ± 0.23	*	*	*	*	ns	ns
A4 (cm^2^)	-0.15 ± 0.26	ns	-	-	-0.32 ± 0.21		-	-	ns	-	-	-	-
A5 (cm^2^)	0.23 ± 0.14	ns	0.33 ± 0.15	*	0.25 ± 0.11	*	0.53 ± 0.16	*	ns	-	-	-	-
A6 (cm^2^)	0.25 ± 0.14	ns	0.32 ± 0.13	*	0.43 ± 0.14	*	0.35 ± 0.17	*	ns	-	-	-	-
TSA (cm^2^)	1.64 ± 0.25	*	-1.14 ± 0.32	*	1.23 ± 0.37	*	-0.66 ± 0.28	*	*	*	*	ns	ns

Δ(T2–T1) values are shown. W: Wilcoxon signed-rank test; KW: Kruskal–Wallis analysis; *: p < 0.05; ns: not significant; (–): not applied; X̅: mean; SE: standard error; TV: total volume; TSA: total surface area. V4/A4 values are not computed in extraction subgroups (first premolars absent).

Evaluation of anterior palatal volume (V3) revealed that treatment-related changes within each group were statistically significant (*p* < 0.05, Wilcoxon test) (Table 2). Kruskal–Wallis analyses confirmed significant intergroup differences (*p* < 0.05), and Mann–Whitney U tests revealed that these differences were driven by extraction versus non-extraction treatment strategies. The V4 volume measurement showed a significant post-treatment increase only in the Class II non-extraction group (Wilcoxon test, *p* < 0.05). However, intergroup comparisons with the Kruskal–Wallis test revealed no significant differences relative to controls. According to the intragroup comparisons, V5 volume increased significantly in both Class II and Class I non-extraction groups (Wilcoxon test, *p* < 0.05), while the reduction observed in the Class II extraction subgroup was not significant. Intergroup comparisons (Kruskal–Wallis) confirmed overall significant differences (*p* < 0.05), again associated with extraction versus non-extraction treatment mechanics.

Treatment changes in total volume (TV) measurements were found to be statistically significant in all groups (*p* < 0.05, Wilcoxon test) (Table 2). Intergroup comparisons were also statistically significant according to the Kruskal–Wallis test (*p* < 0.05), and Mann–Whitney U test results revealed that these differences primarily originated from extraction versus non-extraction treatment strategies (*p* < 0.05).

In terms of intragroup comparisons (Table 2), the anterior surface (A3) and total palatal surface areas (TSA) increased significantly in all groups (Wilcoxon test, *p* < 0.05). Intergroup comparisons also indicated statistically significant differences (Kruskal–Wallis, *p* < 0.05), with pairwise Mann–Whitney U analyses showing that these differences originated mainly from extraction versus non-extraction approaches.

Multiple linear regression analyses revealed that sagittal dentoalveolar changes were independently associated with three-dimensional palatal remodeling (Table 3).

**Table 3:** Multivariable linear regression models assessing the independent associations between treatment-related dentoalveolar changes and changes in total palatal volume (ΔTV) and total palatal surface area (ΔTSA).

	ΔTotal palatal volume (cm^3^)		ΔTotal palatal surface area (cm^2^)	
	β	p	β	p
Independent variable				
ΔU1 / NA (°)	0.06	0.732	-0.154	0.342
ΔU1—NA (mm)	-0.004	0.985	0.119	0.554
ΔU1⊥VRL (mm)	0.361	0.068	0.459	0.013
ΔU6⊥VRL (mm)	-0.353	0.002	-0.386	< 0.001
ΔU1⊥SN (mm)	0.223	0.035	0.16	0.099
ΔU6⊥SN (mm)	-0.073	0.501	-0.146	0.144
Covariates				
Treatment duration (months)	-0.062	0.57	-0.006	0.952
Baseline palatal measure (T1)	0.059	0.552	-0.106	0.261
SN—GoGn (°)	0.149	0.136	0.174	0.063
Model statistics				
F (p value)	8.00 (p < .001)		7.80 (p < .001)	
R^2^ / Adjusted R^2^	0.463 / 0.436		0.543 / 0.498	
ΔR^2^ (p value)	0.027 (ns)		0.046 (ns)	

β: standardized regression coefficients; p < 0.05: statistically significant; Δ: T2–T1 changes; F (p): Overall model significance; R^2^: Total proportion of variance in the dependent variable explained by the full regression model; Adjusted R^2^: R^2^ adjusted for the number of predictors included in the model; ΔR^2^ (p): Change in explained variance after adding covariates to the model; ns indicates non-significant contribution of covariates.

For changes in total palatal volume (ΔTV), sagittal displacement of the maxillary central incisors relative to the vertical reference line (ΔU1⊥VRL) showed a positive trend toward volumetric increase, although this association did not reach statistical significance (*p* = 0.068). In contrast, posterior displacement of the maxillary first molars (ΔU6⊥VRL) was significantly and negatively associated with changes in total palatal volume (*p* < 0.05). Vertical displacement of the maxillary incisors relative to the SN plane (ΔU1⊥SN) was also significantly associated with changes in ΔTV, while angular incisor inclination (ΔU1/NA) and sagittal position relative to the NA line (ΔU1⊥NA) were not significant predictors.

Similarly, regression analysis for total palatal surface area changes (ΔTSA) demonstrated that sagittal movements of the maxillary incisors (ΔU1⊥VRL) and molars (ΔU6⊥VRL) were the primary predictors of surface-area remodeling (*p* < 0.05). Vertical dental changes did not retain statistical significance in the surface-area model. Treatment duration, baseline palatal measurements, and vertical skeletal pattern (SN–GoGn°) did not show independent associations with either ΔTV or ΔTSA. The regression models were statistically significant overall, explaining a substantial proportion of variance in both volumetric and surface-area outcomes (R^2^ and adjusted R^2^ values shown in Table 3).

## Discussion

Treatment-related changes in late adolescent and young adult patients treated with extraction or non-extraction protocols primarily involve dentoalveolar structures, while skeletal and soft-tissue effects remain limited. Recent evidence indicates that palatal morphology, traditionally considered a passive structure, may undergo adaptive remodeling in response to dentoalveolar movements and orthodontic mechanics^10,23^. However, most previous studies relied on conventional or two-dimensional assessments, limiting the evaluation of volumetric and surface-area adaptations. With the advent of digital orthodontics, three-dimensional (3D) model analysis enables precise and reproducible quantification of palatal morphology, providing more detailed insight into treatment-related morphological adaptations associated with different orthodontic strategies.

The inclusion of both U1⊥NA and U1⊥VRL was designed to account for potential A-point remodeling associated with extraction treatments; therefore, VRL was additionally used as a more stable sagittal reference to evaluate maxillary incisor position^24^. Sagittal positional changes of the maxillary incisors, assessed by linear measurements (U1⊥NA, U1⊥VRL), showed significant protrusion only in the Class I non-extraction group and significant retrusion in the Class I extraction group (Wilcoxon, p < 0.05). Intergroup differences confirmed that these positional changes were primarily associated with the applied treatment mechanics (Kruskal–Wallis, Mann–Whitney U; Table 2)^25^. Changes in mandibular incisor inclination (L1/NB) were predominantly observed in the non-extraction groups and are consistent with expected dentoalveolar responses to fixed orthodontic mechanics, including crowding relief and leveling procedures. Literature suggests approximately 0.5° angular and 0.2 mm linear incisor protrusion per millimeter of crowding eliminated, which aligns closely with the present findings^26,27^.

These alterations represent routine treatment-related dentoalveolar adaptations rather than variables directly associated with three-dimensional palatal surface area or volume changes. Accordingly, mandibular incisor inclination was interpreted as a secondary dentoalveolar finding and was not considered a primary predictor of palatal morphological remodeling.

Beyond group-based comparisons, multivariable regression analyses provided insight into the mechanisms underlying palatal remodeling. The results demonstrated that sagittal dentoalveolar movements—rather than isolated angular or vertical dental changes—were independently associated with treatment-related changes in palatal morphology. Specifically, sagittal displacement of the maxillary central incisors (U1⊥VRL) was significantly associated with increases in total palatal surface area (TSA) and showed a borderline positive association with total palatal volume (TV), suggesting that anterior sagittal repositioning may facilitate transverse and volumetric palatal adaptation.

Conversely, posterior displacement of the maxillary first molars (U6⊥VRL) was negatively associated with both volumetric and surface-area outcomes, likely reflecting anchorage-related mechanics and posterior space consumption that may constrain palatal adaptation. In contrast, angular incisor inclination (U1/NA) and vertical dental movements (U1⊥SN, U6⊥SN) did not retain independent predictive value once sagittal movements were accounted for. Although anchorage was not analyzed as a separate categorical variable, its biomechanical influence was inherently captured through sagittal maxillary molar displacement, reflecting anchorage demand and anchorage loss during orthodontic space management.

In the present study, treatment-induced vertical changes of the maxillary incisors (U1⊥SN) showed similar trends across all groups (Table 2). Previous reports have described pseudo-extrusion during space closure, attributed to angular reduction accompanying incisor retraction^28^, and residual post-pubertal dentoalveolar development may also contribute to vertical changes^29^. In the multivariable analyses, U1⊥SN retained an independent association with changes in total palatal volume, whereas no independent association was observed for total palatal surface area, indicating that vertical incisor displacement may contribute to volumetric remodeling but does not primarily explain surface-area changes. Consistent with prior findings reporting no major vertical differences between extraction and non-extraction protocols^30,31^, these results suggest that palatal remodeling is driven predominantly by sagittal space redistribution rather than by isolated angular or vertical dental movements.

In the present study, sagittal evaluation of the maxillary first molars (U6D⊥VRL) revealed significant distalization in the Class II non-extraction group and mesialization in both Class I and Class II extraction groups (Table 2). This result suggests that space gain in non-extraction cases was achieved through distalization; whereas mesialization in extraction cases may be associated with the moderate anchorage or anchorage loss. Generally, extraction spaces are closed by incisor retraction and/or molar mesialization at varying rates depending on the anchorage requirements. However, in non-extraction case, changes in the sagittal positions of the molars in the direction of distalization are expected depending on the type of malocclusion and the mechanics applied^32,33^.

Differently from our study, Hayasaki et al. reported that in both Class I and Class II individuals with similar ages, maxillary first molars exhibited mesialization across all groups, which was more pronounced in extraction cases. The researchers suggested that, in extraction groups, this finding may be associated with anchorage loss, whereas in non-extraction groups, it could be attributed to varying amounts of distalization depending on the severity of the malocclusion and the ongoing growth and development process^34^.

In this study, although numerical increases in molar extrusion were observed in all subgroups (with/without extraction) of Class I and Class II malocclusion groups, no statistically significant differences were found between the groups. In this respect, the results obtained in this study are in line with those of other studies^30^. It is reported that Molar extrusion may occur as a secondary effect of intermaxillary elastics or mechanical side forces during fixed orthodontic treatment^35^. Given that vertical growth of the dentofacial complex can continue into late adolescence, the minor vertical increases observed in our late adolescent cohort may partly reflect ongoing alveolar development^36^. However, in the multivariable regression analyses, vertical displacement of the maxillary first molars (U6⊥SN) did not demonstrate an independent association with treatment-related changes in total palatal volume or surface area, indicating that molar extrusion alone does not play a primary role in three-dimensional palatal remodeling.

In this study, palatal morphology was evaluated using ten distinct parameters, comprising five volumetric and five surface-area measurements. To ensure the interpretability of these segment-based estimates, we verified intra-examiner reliability; excellent agreement (ICCs ≥ 0.90) supported the precision of the proposed reference planes and segmentation workflow (Supplementary Table S1). Considering that palatal volume measurements may be influenced by root inclinations, the horizontal reference plane was defined using the gingival cervical points of the teeth rather than the occlusal plane to minimize geometric distortion in the transverse dimension.

Although fixed orthodontic treatment primarily targets dentoalveolar changes, it is well known that soft tissues also adapt to these changes. In addition, it has been noted that morphological differences may occur in the palatine bone due to both environmental factors and orthodontic forces^9,13^. Previous studies in adults reported no significant differences in total palatal volume or surface area between Class I and Class II malocclusions, despite individual variability in palatal height and width^12^. In our study, consistent with the previous study, the total palatal volume (TV) and total surface area (TSA) values at pre-treatment were found to be similar between groups. However, when the extraction and non-extraction subgroups were compared within in each malocclusion category, significant baseline differences were observed in area and volume measurements, particularly in the anterior (V3, A3) and first premolar (V4, A4) segments (Table 1). This difference can be said to be related to the amount of arch length discrepancy in the anterior region. It should be noted that the absence of statistically significant baseline differences does not imply equivalence between groups, and baseline palatal dimensions may still influence post-treatment values.

In our study, the increases in anterior volume (V3) and anterior surface area (A3) after treatment were found to be statistically significant in all groups (Wilcoxon, *p* < 0.05; Table 2). The fact that this increase was similar in treatment groups that received the same treatment strategy (with or without extraction) indicates that the differences were essentially due to the extraction strategy. Indeed, the greater increase in anterior volume observed in the extraction groups supports the findings of a previous similar study^9^. The increases observed in V3 and A3 measurements during extraction treatments may be associated with retroclination of the maxillary incisors (U1/NA), posterior repositioning of labially displaced canines, and the accompanying resolution of anterior crowding. In support of this interpretation, maxillary incisor inclination showed a significant reduction in the extraction groups, with mean decreases of − 2.62 ± 1.64° in the Class II extraction group and − 3.29 ± 1.67° in the Class I extraction group (*p* < 0.05), concomitant with the observed increases in anterior palatal surface area (A3) and volume (V3). Collectively, these adjustments lengthen the dental arch and enlarge both anterior surface area (A3) and volume (V3). In addition to the vertical position changes observed in the incisors, residual dentoalveolar development continuing into the post-pubertal period may have contributed positively to the increases in volume and area measurements^36^.

In the extraction subgroups of Class I and Class II malocclusions, assessment of first premolar segment volume (V4) and area (A4) was not feasible due to premolar extraction; therefore, these parameters were evaluated only in the non-extraction subgroups.The results demonstrated comparable treatment-related changes in V4 and A4 between non-extraction Class I and Class II groups, indicating a similar adaptive response within the anterior–posterior transition zone.

The volume (V5) of the second premolar region showed a significant increase with treatment only in the non-extraction groups, while no significant change was detected in the extraction groups. In contrast, the surface area of this region (A5) increased significantly in both Class I and Class II extraction groups, and also in the Class I non-extraction group (Table 2; Wilcoxon). These findings suggest that the observed increases are associated with morphological adaptation following the elimination of residual crowding in the buccal segments. This interpretation aligns with Heiser et al., who reported comparable transverse adaptation of the palatal vault after space redistribution^10^.

Although changes in molar region volume measurement (V6) following treatment were significant only in the Class I non-extraction group, intergroup differences were not statistically significant. This relative stability may be attributed to the limited space available for morphological change in the molar region.

Although A6 measurements showed numerical increases across all groups, statistically significant changes were observed primarily in the Class II extraction group and in both extraction and non-extraction subgroups of Class I. However, intergroup comparisons did not reveal significant differences in A6 changes, indicating a comparable magnitude of change across groups. This pattern was consistent with the findings observed for V6 measurements.

In the present study, total palatal volume (TV) showed a significant increase from pretreatment to post-treatment in the non-extraction Class I and Class II groups, whereas significant reductions in both total palatal volume and surface area were observed in the corresponding extraction groups. These contrasting patterns indicate that palatal morphology undergoes different modes of remodeling depending on extraction versus non-extraction treatment approaches. The volumetric increases observed in non-extraction groups may reflect palatal dimensional expansion accompanying arch development and distalization, whereas the reductions observed in extraction groups may be related to the compacting effects of sagittal space-closure mechanics on palatal morphology. Similar trends have been reported by Heiser et al.^9^.

These volumetric changes should be interpreted with caution, as the present study was limited to morphological assessment of palatal adaptations. Although previous studies have reported associations between extraction mechanics and changes in intraoral spatial conditions^20,21^, the current analysis was not designed to directly evaluate functional outcomes. Nevertheless, multivariable regression analyses demonstrated that changes in palatal volume and surface area were primarily associated with sagittal dentoalveolar movements. In particular, sagittal displacement of the maxillary incisors represents a linear movement that directly influences dental arch length and anterior palatal space, independent of angular changes, and was positively associated with increases in palatal volume. In addition, posterior displacement of the maxillary first molars—as reflected by decreasing U6⊥VRL values—was associated with increases in palatal volume and surface area. These findings suggest that the observed palatal changes should be interpreted as morphological adaptations related to applied sagittal treatment mechanics rather than as direct indicators of functional outcomes. From a clinical perspective, the results highlight the importance of anchorage management and sagittal mechanics in cases requiring extractions, while standardized three-dimensional palatal measurements may provide an objective tool for monitoring treatment-related morphological changes.

In the present study, although premolar extraction would theoretically be expected to reduce total palatal volume by approximately 0.97 cm^3^—corresponding to the mean pretreatment volume of the first premolar segment (V4; Table 1)—the actual volumetric reductions observed at the end of treatment were more limited, amounting to − 0.57 cm^3^ in the Class II group and − 0.37 cm^3^ in the Class I group. This difference appears to be related to compensatory volumetric gains in the anterior palatal region (V3), where canine repositioning and maxillary incisor retroclination contributed to an effective enlargement of the anterior palatal surface.

Moreover, premolar extractions have been reported to influence oral cavity dimensions and intraoral spatial relationships in previous CBCT-based studies^20,37^, which may be relevant to broader functional considerations. However, tongue posture and airway volume were not directly evaluated in the present study; therefore, any functional implications should be interpreted cautiously and regarded as indirect. In this context, the current findings primarily reflect treatment-related palatal remodeling rather than definitive functional adaptations. Reports describing adaptive alterations in palatal morphology following extraction therapy support the observed morphological trends^20,36^.

Overall, the results did not support the null hypothesis, as significant differences in dentoalveolar parameters and three-dimensional palatal morphology were observed between extraction and non-extraction treatment protocols. These findings indicate that palatal morphological outcomes vary in association with orthodontic space-management strategies in post-pubertal patients.

The present findings demonstrate that orthodontic treatment strategies (extraction vs non-extraction) exert a measurable and clinically relevant influence on palatal morphology. Non-extraction protocols were associated with increases in palatal volume and surface area, whereas extraction treatments showed corresponding reductions, reflecting differences in applied space-management mechanics. In non-extraction approaches, distalization, incisor protrusion, and transverse arch development may contribute to increased palatal dimensions, whereas in extraction protocols, space-closure mechanics and incisor retraction may be associated with reduced palatal volume and surface area. However, as specific biomechanical variables were not directly quantified, these interpretations should be considered explanatory rather than causal.

Importantly, the results indicate that palatal morphology adapts alongside dentoalveolar movements during orthodontic treatment rather than remaining unchanged. Accordingly, palatal remodeling should be interpreted in relation to applied treatment mechanics and space-management strategies. From a treatment-planning perspective, careful anchorage control and transverse management may help limit treatment-related reductions in palatal volume in extraction cases. Incorporating standardized three-dimensional digital palatal measurements into routine orthodontic records provides an objective framework for evaluating treatment-related morphological changes and complements conventional dental and skeletal assessments.

## Limitations

This single-center retrospective study with a modest sample size may limit generalizability and reduce power to detect small between-group effects. Although all participants were post-pubertal, residual alveolar development could have influenced vertical and volumetric outcomes.

The cohort comprised post-pubertal Turkish patients treated with standardized fixed-mechanotherapy protocol; thus, findings may not be generalizable to growing patients, other ethnicities, or alternative mechanics (e.g., TAD-assisted protocols, clear aligners).

Although intra-examiner reliability was excellent (ICC ≥ 0.90), inter-examiner reliability was not assessed. Palatal morphology was derived from digital casts using predefined reference planes; while this avoided additional radiation, it did not account for soft-tissue elasticity or dynamic functional factors such as tongue posture, neutral-zone mapping, or airway dynamics. Accordingly, any functional interpretations should be considered indirect.

In the extraction subgroups, the absence of first premolars limited segmental comparability in the V4/A4 region. Baseline differences (e.g., arch-length discrepancy) and unmeasured confounders inherent to retrospective designs (e.g., anchorage level, elastic wear or patient compliance) may also have influenced outcomes despite standardized mechanics, although anchorage-related effects were indirectly reflected by sagittal maxillary molar displacement.

Despite these limitations, the use of standardized digital reference planes and 3D model segmentation enabled a reproducible quantitative assessment of palatal morphology. This methodological approach may serve as a robust basis for future validation studies. Further research should focus on multicenter, prospective cohorts with standardized treatment mechanics and long-term follow-up to assess stability and relapse. The inclusion of objective functional measures and integrated skeletal and soft-tissue 3D imaging may provide additional insight into palatal adaptations.

## Conclusions

In post-pubertal Class II and Class I patients treated with fixed camouflage protocols, skeletal changes were limited, whereas dentoalveolar and three-dimensional palatal responses varied according to extraction and non-extraction approaches. Overall, palatal surface area and volume demonstrated distinct remodeling patterns in relation to different orthodontic space-management strategies, indicating that palatal morphology may adapt alongside dentoalveolar changes during treatment. These findings underscore the clinical value of integrating standardized three-dimensional digital cast analysis with conventional cephalometric assessment to characterize treatment-related morphological differences. Although functional parameters were not directly assessed, three-dimensional palatal metrics provide a reproducible framework for comparative evaluation of treatment-related morphological patterns.

## Methods

This retrospective comparative study was approved by the Ankara University Faculty of Dentistry Clinical Research Ethics Committee (15.09.2021; No. 36290600/58/2021). and conducted in accordance with the principles of the Declaration of Helsinki and relevant institutional guidelines and regulations. The study adhered to the Strengthening the Reporting of Observational Studies in Epidemiology (STROBE) guidelines.

The sample was drawn from the archives of the Department of Orthodontics, Ankara University Faculty of Dentistry. The sample comprised post-pubertal Turkish males and females with skeletal or dental Class I/II malocclusions who had undergone fixed mechanotherapy with pre-adjusted edgewise appliances (2010–2019). Individuals with transverse deficiency, congenitally missing teeth, a history of clefts, dentofacial deformities, or syndromes were excluded. Written informed consent for the academic use of medical records was obtained from all participants at the beginning of treatment as part of routine clinical procedures, and all data were anonymized prior to analysis.

### Sample size and grouping

Power analysis was conducted using G*Power 3.1 software (Heinrich Heine University Düsseldorf, Düsseldorf, Germany). Assuming an effect size of 0.70, a significance level of 0.05, and a power of 0.80, the minimum required sample size was calculated as 10 subjects per group to detect differences between pre- and post-treatment measurements. Accordingly, with four groups, the total minimum sample size was determined to be 40.

A total of 500 patient records were screened from the institutional archives. After applying the inclusion and exclusion criteria, 69 post-pubertal individuals were included and allocated to Class II extraction and non-extraction treatment groups and Class I extraction and non-extraction control groups. The final sample size (n = 69) exceeded the a priori required minimum (n = 40) calculated in G*Power; therefore, under the same assumptions (α = 0.05, effect size = 0.70), the study retained at least the planned power of 0.80 for the primary pre–post comparisons.

Skeletal classification was based on ANB° angle measurements: individuals with an ANB = 0°- 4° were assigned to the Class I control group, whereas those with an ANB > 4° were allocated to the Class II treatment group.

Inclusion criteria included permanent dentition with fully erupted second molars, normodivergent growth pattern, and availability of high-quality standardized records at both pretreatment (T1) and post-treatment (T2). Patients with crossbites, open bites, maxillary diastema, missing teeth, prior orthodontic treatment, or systemic disease/syndrome were excluded.

Additional eligibility/quality criteria for the study was determined as follows:

- Model quality: absence of broken/defective teeth on casts; no voids, bubbles, or fractures that distort palatal soft-tissue contours; both T1 and T2 maxillary casts present and scannable; palatal reference landmarks (incisive papilla, interdental papillae, gingival margins) clearly discernible. Cases with missing or poor-quality models at either time point were excluded.
- Radiograph quality: standardized lateral cephalograms available at T1 and T2, free of motion artifact and distortion, with reproducible head positioning; studies lacking adequate radiographs were excluded.
- Treatment completeness: only patients who completed comprehensive fixed-appliance therapy with debonding and documented end-of-treatment records were included; interrupted/abandoned or prematurely terminated treatments were excluded.

*Experimental group:* The study group consisted of 25 females and 9 males in the post-pubertal period with skeletal Class II malocclusion, who were divided into two subgroups: extraction cases treated with first-premolar extractions (bimaxillary or maxillary-only) (n = 16; 9 females, 7 males) and non-extraction cases (n = 18; 16 females, 2 males).

*Control group:* The control group comprised 20 females and 15 males in the post-pubertal period with skeletal Class I malocclusion, divided into two subgroups: extraction cases treated with first-premolar extractions (bimaxillary or maxillary-only) (n = 17; 9 females, 8 males) and non-extraction cases (n = 18; 11 females, 7 males).

For both the study and control groups, subgroup allocation was performed by selecting individuals with similar age, sex and maxillary arch length discrepancies, ensuring homogeneity between the groups and minimizing potential bias. Post-pubertal status was verified on hand–wrist radiographs. Skeletal age was estimated using the Greulich–Pyle Atlas (1959), and maturation stage was classified according to Hägg & Taranger; only subjects at or beyond the DP3u indicator were eligible. For baseline comparisons, skeletal maturation was expressed as a percentage (SM%) by mapping Greulich–Pyle bone age to sex-specific adult standards (higher values indicate more advanced maturation).

### Orthodontic treatment and records

All patients received comprehensive fixed mechanotherapy with 0.018″ slot, Roth-prescription pre-adjusted edgewise brackets under a unified department protocol. Leveling and alignment were performed with NiTi round archwires, followed by rectangular stainless-steel (SS) working wires (up to 0.016 × 0.022″).

In extraction cases, spaces were closed using sliding mechanics on rectangular SS wires with elastomeric chain or NiTi closed-coil springs and conventional posterior dental anchorage without headgear or temporary anchorage devices (TADs). Intermaxillary elastics (e.g., Class II/finishing) were used as needed. No extraoral appliances, temporary anchorage devices, expanders, or adjunctive accelerated-tooth-movement procedures (e.g., vibration, corticotomy, laser) were employed. Treatment end-points included well-aligned arches, acceptable overjet and overbite, stable intercuspation, and achievement of the planned sagittal relationships.

For each patient, records at T1 (pretreatment) and T2 (post-treatment, at debonding) included lateral cephalometric and panoramic radiographs, dental casts or digital models, and treatment charts.

### Cephalometric analysis

Skeletal and dentoalveolar measurements at the pre-treatment (T1) and post-treatment (T2) stages were traced in Dolphin Imaging® (Chatsworth, CA, USA). In addition to conventional measurements, a Vertical Reference Line (VRL) was defined as the line perpendicular to the Sella–Nasion (SN) plane through Sella to quantify sagittal positions of the maxillary incisors and first molars. The specific cephalometric landmarks and linear/angular measurements are provided in detail in Supplementary Methods S1.

### Digital model acquisition and palatal measurements

All dental models obtained from impressions taken with alginate in the pre-treatment (T1) and post-treatment (T2) stages were scanned using the Sirona inEos X5 surface scanner (Sirona inEos X5, Dentsply Sirona, Bensheim, Germany) and converted into digital models using the Inlab CAD SW 16.0 Basic Module (Dentsply Sirona, Bensheim, Germany), and all measurements were performed on the digital models. Arch length discrepancy (ALD, mm) was calculated on pretreatment (T1) digital maxillary models using 3Shape OrthoAnalyzer (3Shape A/S, Copenhagen, Denmark) as arch perimeter minus required tooth material (first molar to first molar); negative values indicate crowding, positive values spacing. For the assessment of palatal morphology, the Materialise 3-Matic 17.01 software (Materialise NV, Leuven, Belgium) was utilized. Accordingly, specific reference points and planes were established on the digital maxillary models, and both surface area and volumetric measurements were obtained.

**Fig. 1:**
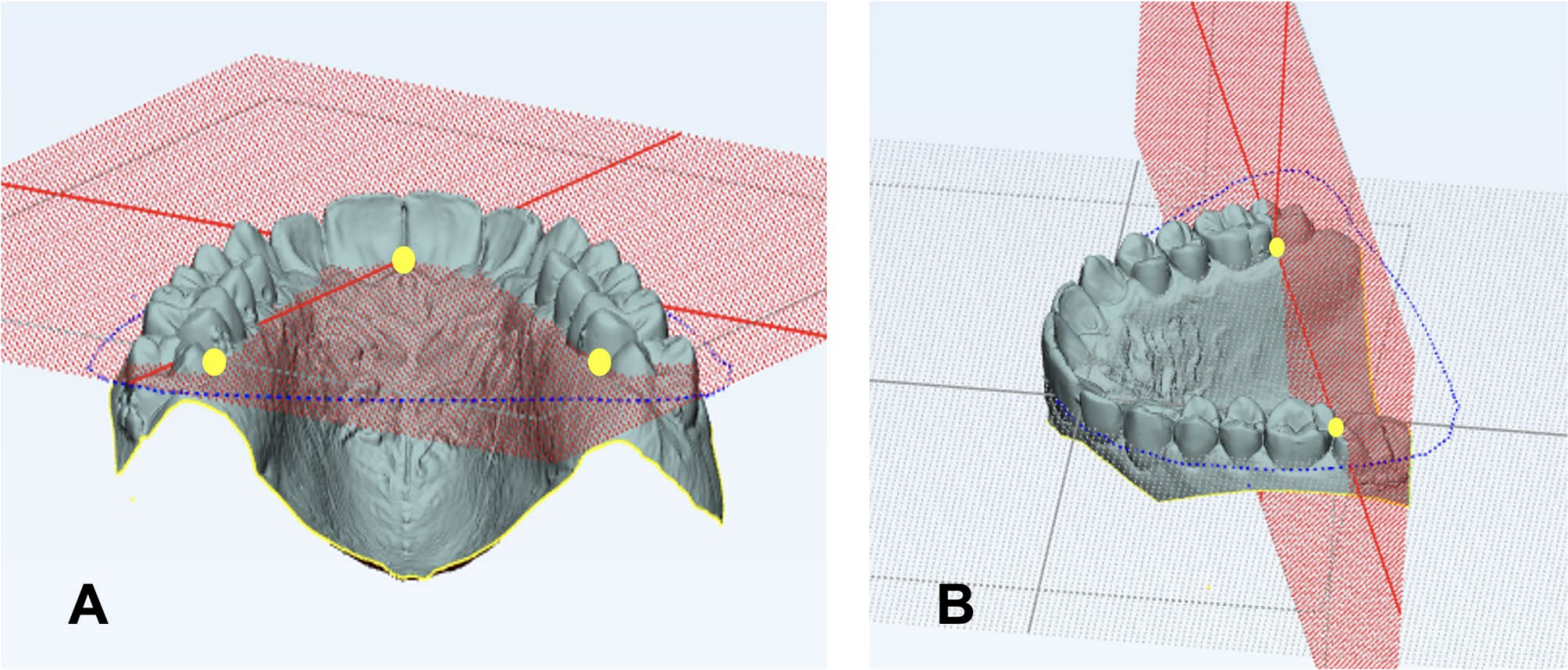
Construction of reference planes on maxillary digital models. (**A**) Horizontal reference plane (HRP): plane formed by the incisive papilla and distal points of the right and left first molars. (**B**) Posterior reference plane (PRP): plane passing through the distal contact points of the right and left first molars, perpendicular to the HRP.

In this context, the most anterior point of the incisive papilla, the distal ends of the palatal interdental papillae up to the first molars, and the deepest cervical points of the dentogingival junctions were identified. Two reference planes were constructed on the maxillary models (Fig. 1): a Horizontal Reference Plane (HRP) defined by the incisive papilla and the distal points of the right and left first molars, and a Posterior Reference Plane (PRP) passing through the distal contact points of the right and left first molars, perpendicular to the HRP.

**Fig. 2:**
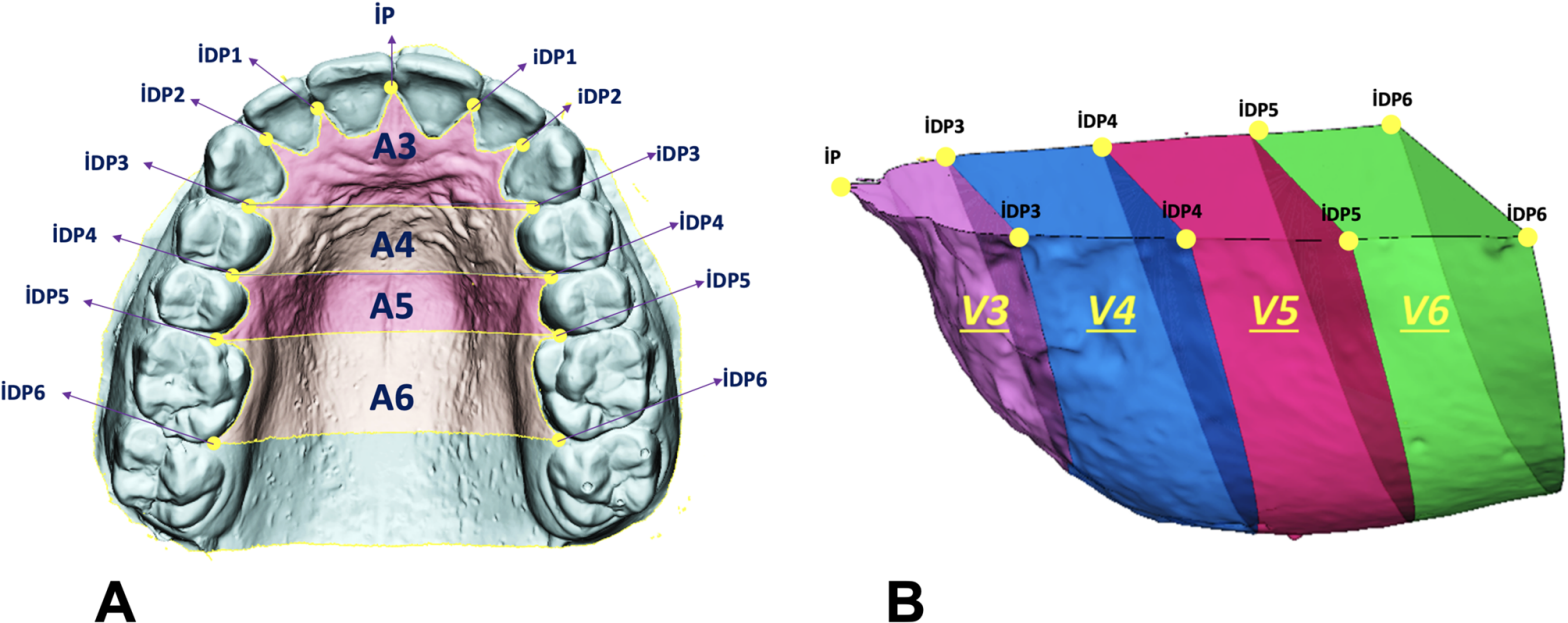
Representation of reference points and segmented palatal surface (A3–A6) and volume (V3–V6) regions on maxillary digital models. IP: Interincisal papilla. iDP1–iDP6: Interdental papilla points between the respective teeth (iDP1: central–lateral incisors; iDP2: lateral–canine; iDP3: canine–first premolar; iDP4: first premolar–second premolar; iDP5: second premolar–first molar; iDP6: first–second molar). (**A**) Palatal surface areas (A3–A6) were defined by planes passing through consecutive interdental papilla points (iDP3–iDP6) and bounded superiorly by the gingival margin. A3 corresponds to the anterior region limited posteriorly by the plane through iDP3 points; A4, A5, and A6 represent successive posterior segments between adjacent interdental planes (iDP3–iDP4, iDP4–iDP5, iDP5–iDP6, respectively). (**B**) Corresponding volumetric regions (V3–V6) were enclosed by the horizontal reference plane and vertical planes passing through the respective interdental papilla points (iDP3–iDP6) down to the palatal mucosa. V3, V4, V5, and V6 denote the anterior-to-posterior volumetric compartments defined by these planes.

On the digital models, five volumetric measurements (cm^3^) were performed within the space bordered superiorly by the horizontal reference plane, inferiorly by the palatal mucosa, and posteriorly by the posterior reference plane (Fig. 2). In addition, five surface area measurements (cm^2^) were obtained on the surface of palatal mucosa, extending superiorly from the gingival margin to the posterior reference plane. The total volume (TV) and total surface area (TSA) measurements were divided into four segments corresponding to the anterior, first premolar, second premolar, and molar regions. These measurements and landmarks are illustrated in Fig. 2. All cephalometric tracings and digital model measurements at T1 and T2 were performed by the same examiner. To assess intra-examiner reliability, 70 randomly selected cephalometric radiographs and their corresponding casts were re-measured 30 days after the initial assessment.

### Statistical analysis

All data analyses were performed using SPSS version 28 (IBM Corp., Armonk, NY, USA). All statistical analyses were tested at a significance level of *p* < 0.05. To assess intra-examiner reliability, a random of 70 cephalometric radiographs and corresponding digital models (including pre- and post-treatment records) was re-evaluated after a 30-day interval. Intraclass correlation coefficients (ICC) with 95% confidence intervals were calculated, and detailed results are provided in Supplementary Table S1. At baseline (T1), intergroup differences were examined using the nonparametric Kruskal–Wallis test to assess group comparability.

For treatment effects, intragroup pre–post changes (ΔT2–T1 within each group) were evaluated with the Wilcoxon signed-rank test.

Intergroup differences in change scores (ΔT2–T1) were first evaluated using the nonparametric Kruskal–Wallis test. When a significant overall difference was detected, predefined pairwise comparisons were performed using the Mann–Whitney U test to specifically assess extraction versus non-extraction contrasts within the Class I and Class II cohorts (1–2, 3–4, 1–3, and 2–4). Because these pairwise analyses were hypothesis-driven and limited in number, no additional formal correction for multiple comparisons was applied.

To evaluate whether dentoalveolar changes independently predicted three-dimensional palatal remodeling beyond group-based comparisons, multiple linear regression analyses were performed. Changes in total palatal volume (ΔTV) and total palatal surface area (ΔTSA) were analyzed as dependent variables in separate models. Independent variables included treatment-related changes in maxillary incisor inclination (U1/NA), sagittal incisor position (U1⊥NA and U1⊥VRL), maxillary molar sagittal displacement (U6⊥VRL), and vertical dental changes (U1⊥SN and U6⊥SN). Treatment duration, baseline palatal measurements at T1 (TV or TSA), and vertical skeletal pattern (SN–GoGn°) were included as covariates in all models to control for potential confounding effects. Anchorage was not included as a distinct categorical variable; instead, its biomechanical influence was indirectly accounted for through sagittal maxillary molar displacement (U6⊥VRL), which captures anchorage demand and anchorage loss during space management.

## Supplementary Information

Supplementary Information 1.

Supplementary Information 2.

## Data Availability

The datasets generated and/or analysed during the current study are available from the corresponding author on reasonable request.

## Abbreviations

V3–V6: Segmental palatal volumes (anterior, 1st premolar, 2nd premolar, molar)
TV: Total Volume
VRL: Vertical reference line
ALD: Arch Length Discrepancy
TD: Treatment Duration
W: Wilcoxon signed-rank test
HRP: Horizontal reference plane
TAD: Temporary anchorage device
SS: Stainless Steel
A3–A6: Segmental palatal surface areas (anterior, 1st premolar, 2nd premolar, molar)
TSA: Total Surface Area
CA: Chronological age
SM: Skeletal maturation
KW: Kruskal–Wallis analysis
ICC: Intraclass correlation coefficient
PRP: Posterior reference plane
CBCT: Cone beam computed tomography
