# Supplementary material for: Three dimensional palatal morphology and dentoalveolar differences after extraction and non extraction treatment in class II malocclusion
Source: Sci Rep. 2026 Jan 30;16:6728. doi: 10.1038/s41598-026-37842-y (PMC12913599; doi:10.1038/s41598-026-37842-y)
Supplement: Supplementary file 1 — Supplementary Information 1. [file 41598_2026_37842_MOESM1_ESM.docx]

**Supplementary Materials**

**Methods S1. Cephalometric landmarks and measurements**


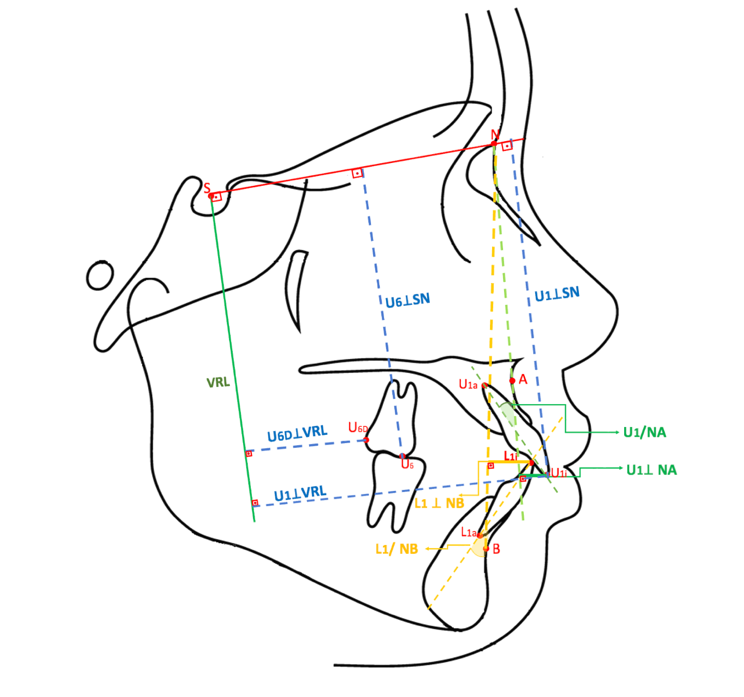

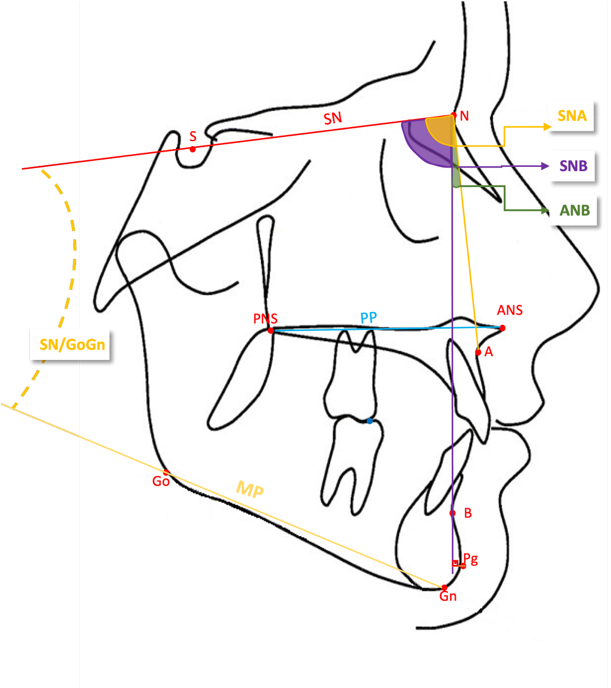


*Cephalometric landmarks*

Sella (S): The geometric midpoint of the sella turcica

Nasion (N): The most superior and anterior point at the junction of the frontal bone and nasal bone on the midsagittal plane

Anterior Nasal Spine (ANS): The most anterior point of the maxilla

Posterior Nasal Spine (PNS): The most posterior point of the palatine bone

Point A (A): The deepest point on the curvature of the maxillary alveolar process

Point B (B): The deepest point on the curvature of the mandibular alveolar process

Pogonion (Pg): The most anterior point of the mandibular symphysis

Menton (Me): The most inferior point at the mandibular symphysis

Gnathion (Gn): The midpoint of the concavity between pogonion and menton on the symphysis

Gonion (Go): The projection of the intersection of the tangent drawn to the posterior border of the ramus and the tangent drawn to the posterior border of the corpus in the gonial region

U1i: The incisal edge of the maxillary central incisor.

U1a: The apex of the maxillary central incisor.

U6: The most prominent point on the mesial cusp of the maxillary first molar.

U6D: The most distal point on the distal crown curvature of the maxillary first molar.

L1i: The incisal edge of the mandibular central incisor.

L1a: The apex of the mandibular central incisor.

Vertical Reference Line (VRL): A line drawn perpendicular to the SN plane passing through Sella, used as the vertical reference for sagittal positional measurements.

*Skeletal measurements*

SNA (°): Maxilla to cranial base (Sella–Nasion–Point A) angle
SNB (°): Mandible to cranial base (Sella–Nasion–Point B) angle
ANB (°): Maxillo-mandibular relationship (difference between SNA and SNB)
SN/GoGn (°): Angle between Sella–Nasion line and mandibular plane (Gonion–Gnathion)

*Dentoalveolar measurements*

L1/NB (°): Mandibular incisor to Nasion–B Point angle

L1⊥NB (mm): Perpendicular distance of mandibular incisor to NB line

U1/NA (°): Maxillary incisor to Nasion–A Point angle

U1⊥NA (mm): Perpendicular distance of maxillary incisor to NA line

U1⊥SN (mm): Perpendicular distance from the incisal edge of the maxillary central incisor to the Sella-Nasion plane

U6⊥SN (mm): Perpendicular distance from the cusp tip of the mesial cusp of the maxillary first molar to the SN plane.

U1⊥VRL (mm): Perpendicular distance from the incisal edge of the maxillary central incisor to Vertical Reference Line

U6D⊥VRL (mm): Perpendicular distance from the distal crown surface/contact point of the maxillary first molar to the VRL.

**Table S1. Intraclass correlation coefficients (ICC) for cephalometric and palatal variables**

| Measurements | ICC |
| --- | --- |
| SNA | 0.92 |
| SNB | 0.91 |
| ANB | 0.94 |
| SN/GoGn | 0.96 |
| U1/NA | 0.92 |
| U1⊥NA | 0.91 |
| L1/NB | 0.91 |
| L1⊥NB | 0.94 |
| U1⊥VRL | 0.92 |
| U6D⊥VRL | 0.96 |
| U1⊥SN | 0.95 |
| U6⊥SN | 0.92 |
| Overjet | 0.95 |
| Overbite | 0.95 |
| V3 | 0.92 |
| V4 | 0.92 |
| V5 | 0.93 |
| V6 | 0.94 |
| TV | 0.94 |
| A3 | 0.92 |
| A4 | 0.93 |
| A5 | 0.95 |
| A6 | 0.97 |
| TSA | 0.94 |

*ICCs ≥ 0.90 indicate excellent intra-examiner reliability. TSA: Total Surface Area; TV: Total Volume.*
